# A tale of 2 realms: spatial processing of the maize phytocytokine Zip1

**DOI:** 10.1093/plphys/kiag649

**Published:** 2026-09-07

**Authors:** Mamoona Khan

**Affiliations:** Assistant Features Editor, Plant Physiology, American Society of Plant Biologists, Rockville, MD 20855, United States; Department of Plant Pathology, Institute of Crop Science and Resource Conservation (INRES), University of Bonn, Nussallee 9, Bonn 53115, Germany

Plants rely on sophisticated cell-to-cell communication to perceive environmental changes and coordinate appropriate responses. Among the signalling molecules involved in these processes are phytocytokines, a diverse group of small, plant-derived peptides that regulate growth, development, and stress responses (Hou et al. 2021; Rzemieniewski and Stegmann 2022). In response to tissue damage or pathogen attack, phytocytokines can act as endogenous danger signals that activate specific receptors and initiate downstream defense responses (Gust et al. 2017).

Many phytocytokines are produced as precursor proteins that require proteolytic processing to generate biologically active signalling molecules. Specific proteases cleave these precursors to generate peptides or peptide-containing fragments that participate in signalling. However, phytocytokines are not processed through a common pathway; different peptide families depend on distinct proteases, cleavage sites, and cellular compartments (Koenig et al. 2026b). While considerable progress has been made in understanding phytocytokine perception and downstream signalling, much less is known about where and how these immune signals are generated and released.

The maize phytocytokine Zea mays Immune Signalling Peptide 1 (Zip1) is an endogenous signalling peptide involved in the regulation of plant immunity. Zip1 is embedded within the precursor protein PROZIP1 and has been linked to salicylic acid–associated immunity and pathogen-induced defense responses (Koenig et al. 2026b). Previous work showed that the papain-like cysteine proteases (PLCPs) CP1 and CP2 contribute to PROZIP1 processing in the apoplast (Koenig et al. 2025; Koenig et al. 2026b). However, how PROZIP1 is initially activated and how the resulting signal reaches the apoplast remained unknown. In a recent issue of *Plant Physiology*, Koenig et al. (2026a) addressed these questions and revealed that PROZIP1 undergoes spatially separated processing inside and outside the cell. Their findings reveal a two-step mechanism that separates phytocytokine activation and release from subsequent signal attenuation.

The authors first asked where PROZIP1 is localized and processed within the cell. Using fluorescently tagged PROZIP1 in maize protoplasts and *Nicotiana benthamiana*, they found that PROZIP1 was predominantly intracellular and partially associated with the endoplasmic reticulum (ER). Mutation of the previously predicted double-arginine (RR) cleavage sites altered PROZIP1 localization. Subcellular fractionation further revealed that a processed C-terminal fragment of PROZIP1 (Ct-PROZIP1) accumulated in the apoplast, whereas mutation of the RR sites strongly reduced this extracellular accumulation. Together, these findings suggest that intracellular processing precedes the release of Ct-PROZIP1 into the apoplast. Interestingly, Ct-PROZIP1 release did not depend on the conventional ER–Golgi pathway, which suggests an alternative route to the apoplast.

The authors next used amino-terminal oriented mass spectrometry (ATOMS) to identify PROZIP1 cleavage sites. They detected several cleavage events after arginine residues, including sites immediately upstream and downstream of the Zip1 region. Notably, cleavage occurred at both paired and single arginine residues. Structural modelling further suggested that several of these residues are exposed on the PROZIP1 surface and therefore accessible to proteases. Together, these findings show that PROZIP1 cleavage is arginine dependent and hint at the type of protease that may be responsible for its intracellular processing.

The arginine-dependent cleavage pattern pointed to metacaspases, a class of proteases known to cleave phytocytokine precursors after basic residues, as potential proteases involved in PROZIP1 processing. Among the 3 type II metacaspases identified in maize, ZmMC9 emerged as the strongest candidate based on its expression and similarity to the Arabidopsis metacaspase AtMC4. Biochemical assays showed that ZmMC9 directly cleaved PROZIP1, with a strong preference for arginine residues. Further cleavage-site analysis revealed that ZmMC9 cuts PROZIP1 at sites flanking the Zip1 region, including cleavage after R84 that generates Ct-PROZIP1. Together, these results show that ZmMC9 can directly process PROZIP1 to generate its C-terminal signalling fragment.

Koenig et al. (2026a) next asked whether Ct-PROZIP1 requires further processing to release free Zip1 or whether the C-terminal fragment itself is biologically active. Surprisingly, delivery of Ct-PROZIP1 into the maize apoplast reduced disease symptoms caused by maize pathogenic fungus *Ustilago maydis* more strongly than free Zip1. This phenotype was abolished when the Zip1 motif within Ct-PROZIP1 was disrupted, showing that the embedded Zip1 sequence is required for its function. These results suggest that Ct-PROZIP1 itself represents a major bioactive form in the apoplast, and is not merely an intermediate in the release of free Zip1.

The finding that Ct-PROZIP1 itself is bioactive raised an important question about the role of the previously identified papain-like cysteine proteases (PLCPs) CP1 and CP2. Therefore, the authors next asked whether the previously identified PLCPs CP1 and CP2 contribute to the initial processing of PROZIP1. Inhibiting intracellular PLCP activity had no effect on PROZIP1 localization or processing, arguing against their involvement in the initial intracellular cleavage. By contrast, recombinant PROZIP1 was readily processed when incubated with apoplastic fluid from maize leaves, and inhibitor assays showed that PLCPs together with other extracellular proteases contribute to this activity. Interestingly, ATOMS analysis mapped extracellular cleavage to sites both flanking and within the Zip1 sequence. This pattern points to a dual role for apoplastic proteolysis: releasing Zip1 from Ct-PROZIP1 while simultaneously promoting its degradation.

Together, the study by Koenig et al. (2026a) reveals that PROZIP1 processing is not simply a mechanism for releasing the mature Zip1 peptide, but a spatially organized process that separates signal activation from attenuation. Intracellular arginine-dependent processing, which can be mediated by the Type II metacaspase ZmMC9, generates the bioactive Ct-PROZIP1 fragment for release into the apoplast. Once outside the cell, Ct-PROZIP1 is exposed to PLCPs and other apoplastic proteases that further process and ultimately restrict the persistence of the signal. This compartmentalization may allow plants to rapidly deploy an immune signal while preventing prolonged activation in the extracellular environment (Fig. 1). However, it remains unclear how Ct-PROZIP1 reaches the apoplast independently of conventional ER–Golgi trafficking. Moreover, the strong bioactivity of Ct-PROZIP1 also raises the question of how this signal is perceived and whether Ct-PROZIP1 and free Zip1 share the same receptor. It also remains to be determined whether this spatial separation of activation and attenuation is unique to PROZIP1 or represents a broader mechanism for controlling phytocytokine signalling.

**Figure 1:**
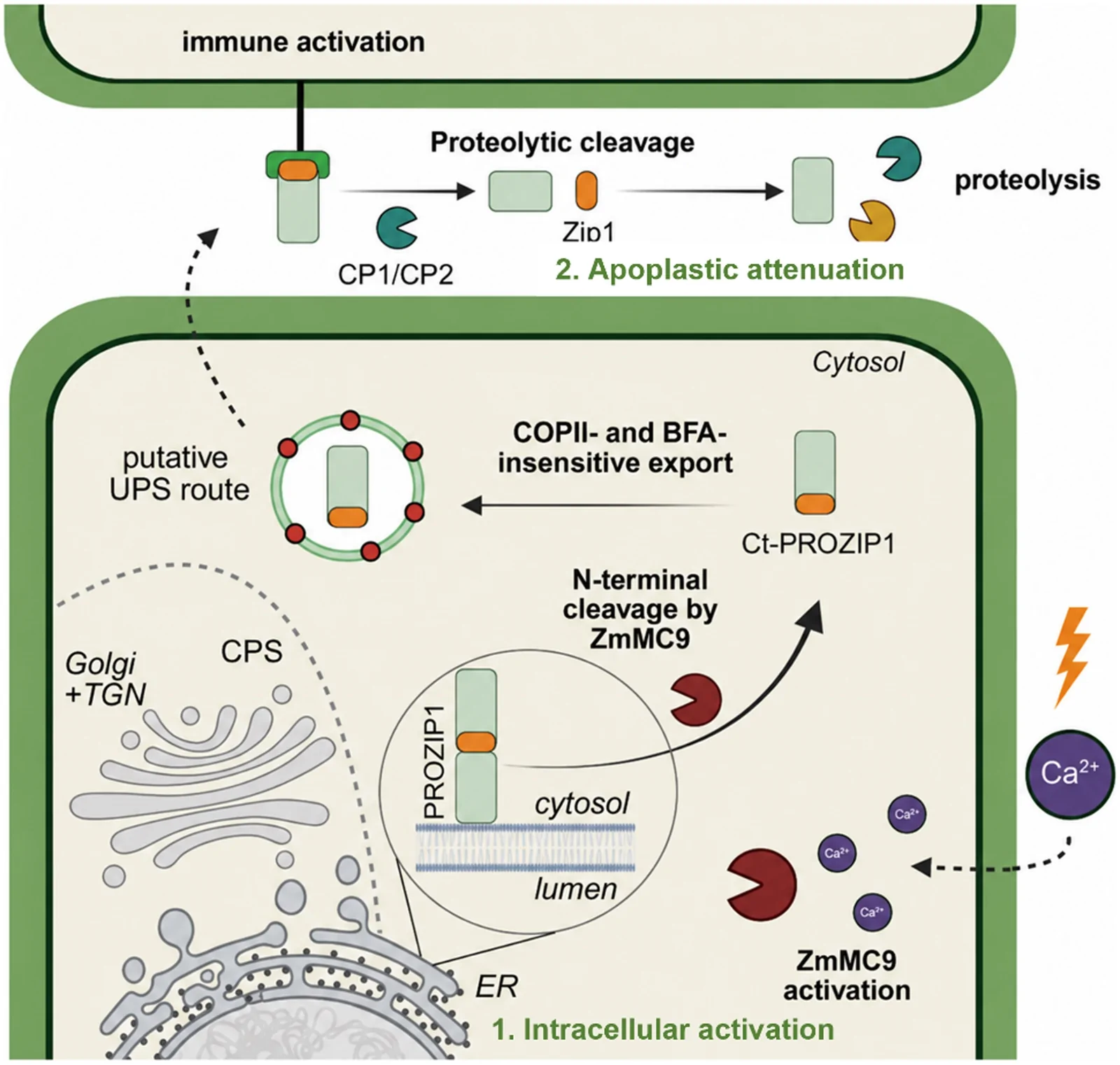
Spatial control of PROZIP1 peptide processing. PROZIP1 undergoes 2 spatially separated proteolytic steps that regulate phytocytokine signalling. (1) Intracellular activation: calcium (Ca^2+^)-dependent ZmMC9 processes PROZIP1 to generate the bioactive C-terminal fragment Ct-PROZIP1, which reaches the apoplast through a putative unconventional secretion route. Ct-PROZIP1 can activate immune signalling, although its receptor remains unknown. (2) Apoplastic attenuation: PLCPs, including CP1 and CP2, together with other apoplastic proteases, further process Ct-PROZIP1. Extracellular cleavage can release free Zip1 but also promotes further peptide degradation, thereby restricting signal persistence (adapted from Koenig et al. 2026a, Fig. 5).

## Recent related articles in *Plant Physiology*

Stuhrwohldt et al. (2020) identified 3 subtilases that control CLE40 maturation through sequential cleavage: one releases the active peptide, while a second attenuates its activity. Pro hydroxylation, addition of hydroxyl group (-OH) to the amino acid proline, prevents this second cleavage and thereby protects bioactive CLE40.

Boschiero et al. 2020 identified 4,439 putative small secreted peptide (SSP)-encoding genes in *Medicago truncatula* and established MtSSPdb as a resource for systematic annotation, and functional characterization of these SSP candidates.

Chen et al. (2026) characterized rice OsSSIP as a pathogen-induced peptide that activates immunity and enhances rice resistance to *Magnaporthe oryzae*. OsSSIP-related peptides also elicited immune responses across monocots and dicots, suggesting broad-spectrum activity.

## Data Availability

No data were generated or analyzed in this study.
